# High-Content Chemical and RNAi Screens for Suppressors of Neurotoxicity in a Huntington's Disease Model

**DOI:** 10.1371/journal.pone.0023841

**Published:** 2011-08-31

**Authors:** Joost Schulte, Katharine J. Sepp, Chaohong Wu, Pengyu Hong, J. Troy Littleton

**Affiliations:** 1 Department of Biology, Department of Brain and Cognitive Sciences, The Picower Institute for Learning and Memory, Massachusetts Institute of Technology, Cambridge, Massachusetts, United States of America; 2 Department of Computer Science, National Center for Behavioral Genomics, Brandeis University, Waltham, Massachusetts, United States of America; National Institutes of Health, United States of America

## Abstract

To identify Huntington's Disease therapeutics, we conducted high-content small molecule and RNAi suppressor screens using a *Drosophila* primary neural culture Huntingtin model. *Drosophila* primary neurons offer a sensitive readout for neurotoxicty, as their neurites develop dysmorphic features in the presence of mutant polyglutamine-expanded Huntingtin compared to nonpathogenic Huntingtin. By tracking the subcellular distribution of mRFP-tagged pathogenic Huntingtin and assaying neurite branch morphology via live-imaging, we identified suppressors that could reduce Huntingtin aggregation and/or prevent the formation of dystrophic neurites. The custom algorithms we used to quantify neurite morphologies in complex cultures provide a useful tool for future high-content screening approaches focused on neurodegenerative disease models. Compounds previously found to be effective aggregation inhibitors in mammalian systems were also effective in *Drosophila* primary cultures, suggesting translational capacity between these models. However, we did not observe a direct correlation between the ability of a compound or gene knockdown to suppress aggregate formation and its ability to rescue dysmorphic neurites. Only a subset of aggregation inhibitors could revert dysmorphic cellular profiles. We identified *lkb1*, an upstream kinase in the mTOR/Insulin pathway, and four novel drugs, Camptothecin, OH-Camptothecin, 18β-Glycyrrhetinic acid, and Carbenoxolone, that were strong suppressors of mutant Huntingtin-induced neurotoxicity. Huntingtin neurotoxicity suppressors identified through our screen also restored viability in an *in vivo Drosophila* Huntington's Disease model, making them attractive candidates for further therapeutic evaluation.

## Introduction

Huntington's Disease (HD) is a dominantly inherited fatal neurodegenerative disorder. It results from expansion of a polygluatamine (polyQ) tract in the Huntingtin (Htt) protein which alters its conformation and function [1]. Neuropathological hallmarks of the disease include Htt aggregation, early-onset striatal neurodegeneration, and late-stage cortical thinning [2], [3]. Mammalian models of HD indicate that neuron-specific dysregulation of cellular physiology contributes to the underlying neuropathology, although subcortical white matter degeneration may also suggest a glial contribution [4]–[7]. Mutant Htt has been suggested to disrupt transcription, proteasome activity, axonal transport, synaptic function, signaling cascades (including the mTOR/Insulin pathway) and other physiological processes in a variety of neuronal subtypes [8].

PolyQ tract length accounts for approximately 70% of the variance in the age-of-onset of Huntington's disease [9], with the remaining variance attributed to disease-modifying agents such as environmental factors or genetic background. Linkage analysis using large affected families is the gold standard for identifying disease modifying loci [10]. However, these investigations require lengthy study periods and considerable resources. *In vitro* genetic or chemical suppressor screens offer another avenue to rapidly identify suppressors. The suitability of cell-based screening models encompasses several considerations. Htt is ubiquitously expressed, yet deleterious effects of mutant polyQ expansion are observed primarily in the central nervous system of HD patients. In conducting suppressor screens, it is therefore desirable to use neuronal systems. Stable neuronal cell lines can change their characteristics over continued passages, and thus may not retain HD phenotypes. In addition, stable cell lines continually divide, which may alter regulation of cell survival and death pathways that are relevant to the human disease state in quiescent neurons. Primary neuronal disease models have many advantages, since cellular growth and differentiation states are more similar to the *in vivo* situation, yet they are amenable to high-throughput chemical and genetic suppressor screens.

Huntingtin is conserved in *Drosophila*, and orthologs of many interacting proteins exist [11]–[13]. Suppressor screening with first generation exon 1 Htt fragments has generated important candidate disease modifying agents [14], [15]. However, protein context has been shown to be an important component of mutant Htt pathogenesis, and therefore there is a need to conduct additional screens using larger Htt constructs in more physiologically relevant systems [16]. To this end, we have developed a *Drosophila* primary neural culture system for HD, using a large human Htt fragment (exons 1–12, 588 amino acids in addition to the polyQ tract). The methodology is suited to high throughput small molecule and RNAi suppressor screening, and offers a desirable balance between physiological relevance and technical tractability.

We demonstrate that a 588 amino acid human Htt fragment containing an expanded polyQ tract (138Q) readily forms cytoplasmic aggregates in primary neurons, in addition to causing aberrant neuronal morphology when compared to non-expanded human Htt controls (15Q). From a screen of approximately 2600 small molecules and a whole genome kinase/phosphatase RNAi library, we identified four new compounds that could revert the mutant phenotype (Camptothecin, 10-Hydroxycamptothecin, 18β-Glycyrrhetinic acid, Carbenoxolone) and knockdown of the *lkb1* kinase, that can suppress polyQ Htt aggregation and revert morphological profiles towards control states. In addition, we identified several previously studied polyQ Htt aggregation inhibitors. This *Drosophila* HD model represents an attractive system for future large-scale suppressor screening. In addition, the current candidates provide new avenues to define pathogenic mechanisms in HD.

## Results

### *Drosophila* Primary Neural Culture Screening System for Huntington's Disease

**Figure 1:**
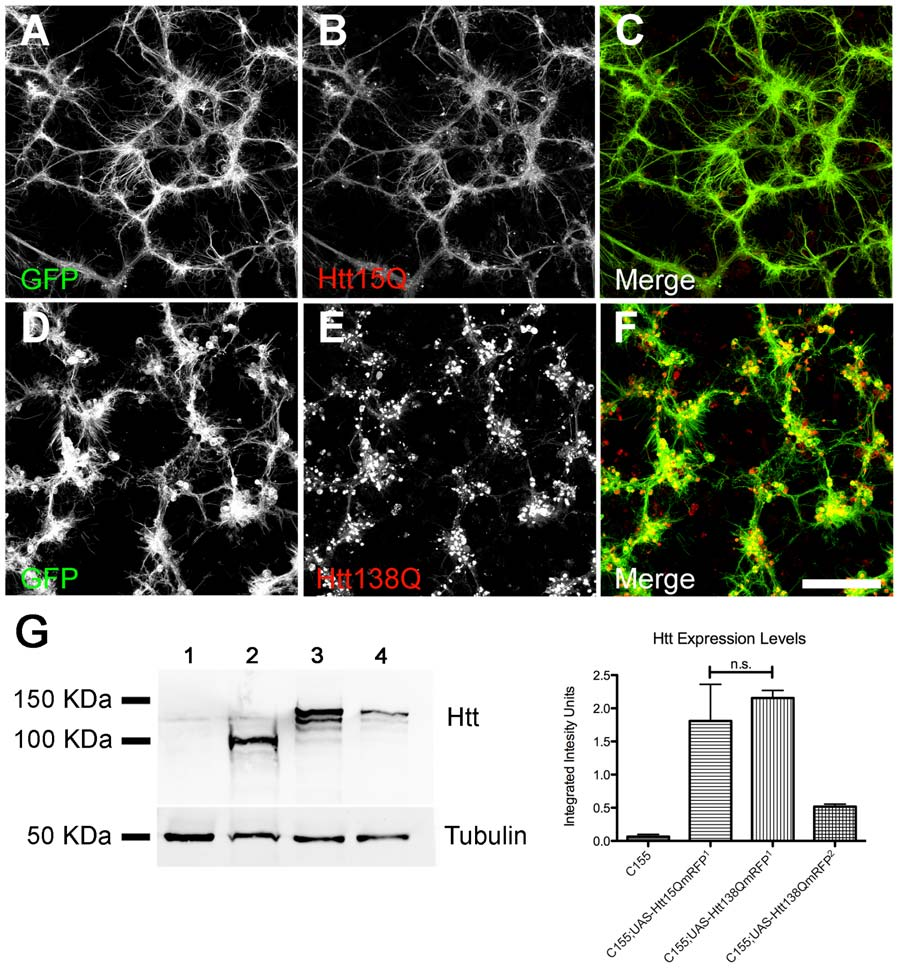
Confocal microscopy images of Htt15Q (A–C) and Htt138Q (D–F) expressing *Drosophila* primary neural cultures plated on glass coverslips. The subcellular distribution of Htt (red channel), morphology of Htt expressing primary cultures (green channel, UAS-CD8GFP), and merged images are shown. Htt15Q (**B**) has a diffuse cytoplasmic distribution and fills most processes of cultured neurons, while Htt138Q forms large insoluble aggregates that accumulate in neurites and within cell bodies of neuromere clusters (**E**). Htt15Q and Htt138Q expressing cultures also display different neuronal morphologies. Htt15Q cultures have long straight neurites (**A**), while Htt138Q cultures have shorter neurites that fail to extend from neuromere clusters leading to a club-like appearance (**B**). (**G**) Quantitative Western blot (n = 4) showing that the relative expression level of Htt15Q^1^ and Htt138Q^1^ is comparable in strains used for primary culture screening (compare lanes 2 and 3). A Htt138Q strain with weaker expression is also shown (lane 4, Htt138Q^2^). The strain genotypes that are listed on the bar graph (left to right) correspond to lanes 1–4 of the blot. Tubulin was used as a loading control. p<0.05. Scale bar: 100 µm.

We have previously described a *Drosophila* Huntington's Disease model which displays many characteristics of human HD, including neurodegeneration, disrupted axonal transport, and decreased longevity [17]. To extend our studies of HD pathology, we generated a new monomeric Red Fluorescent Protein (mRFP) N-terminal tag variant for *in vivo* imaging of Htt distribution (Htt-RFP). The Htt-RFP construct encompasses the caspase-6 cleavage fragment important for Htt toxicity [18] and includes either a non-pathogenic (15Q) or pathogenic (138Q) polyQ tract. This fragment corresponds to exons 1–12 of human Htt and is 588 amino acids in length (∼80 kDa), excluding the polyQ domain and RFP tag. For our studies, we used the GAL4/UAS system [19] to drive expression of the constructs in the nervous system using the pan-neuronal GAL4 driver *Elav^c155^* (*C155*). We selected *UAS-Htt15QmRFP* and *UAS-Htt138QmRFP* strains that had comparable expression levels (i.e. Htt15Q^1^ and Htt138Q^1^) when crossed to *C155* as demonstrated by quantitative Western blotting (Figure 1G). Prominent bands of ∼109 kDa and ∼125 kDa were observed for the Htt15Q^1^ and Htt138Q^1^ strains, respectively, in agreement with the predicted molecular weights of the RFP-fusion proteins. Pan-neuronal expression of *Htt138Q^1^* using C155 causes pupal lethality, while *Htt15Q^1^* controls are viable and have normal longevity (see detailed analysis below). For downstream behavioral analysis, we selected an additional UAS-Htt138QmRFP strain (Htt138Q^2^) that has reduced Htt138Q protein expression (Figure 1G) and is adult viable. The decreased longevity observed in the *Htt138Q^1^* strain is more severe than that observed in our earlier studies [17] and may be related to an increased polyQ length in the new construct (138Q vs. 128Q), a larger Htt N-terminal fragment (588 a.a. vs. 548 a.a.), or differences in Htt expression levels. The severity of the *Htt138Q^1^* allele suggests that this model may correspond to juvenile-onset HD observed in humans [20]. In juvenille-onset HD, the CAG repeats often exceed 55 and phenotypes develop prior to adulthood.

To conduct a small molecule and RNAi screen to identify suppressors of Huntington toxicity we prepared primary neuronal cultures from control (*C155;Htt15QmRFP^1^*, abbreviated as *Htt15Q*) and mutant (*C155;UAS-Htt138QmRFP^1^*, abbreviated as *Htt138Q*) Huntingtin strains that simultaneously expressed membrane associated-GFP (*UAS-CD8GFP*) in all neurons. This dual labeling approach enabled us to track the subcellular distribution of mRFP-tagged Huntingtin, while simultaneously monitoring the general morphology of cultured neurons (Fig. 1 A–F). Visualization of Htt-RFP localization demonstrated that Htt138Q readily forms aggregates which accumulate in cell bodies and neurites, while Htt15Q is soluble and has a more uniform cytoplasmic distribution (Fig. 1, compare 1B, 1E). In addition, we found that Htt138Q-expressing neurons display morphological indicators of reduced neuronal health [21], [22], including smaller neuromeres, increased branching, and reduced axonal connectivity, as monitored by membrane associated-GFP (Fig. 1, compare 1A, 1D). Neurite morphology and Htt aggregation were quantified in cultures plated in 384-well format using custom algorithms developed to process digital images collected via automated microscopy [23]. Population analysis of Htt15Q and Htt138Q replicate wells revealed that eight morphology features (small, medium, large, and average neuromere size, and short, medium, long, and average neurite length) provided robust data content to generate effective separation of Htt15Q control from Htt138Q mutant neuronal morphology. Differences in Htt aggregation were also readily detectable between mutant and control cultures using these algorithms. To screen for suppressors of HD toxicity we therefore monitored the presence of Htt aggregates, as well as morphology, to evaluate overall neuron health.

### Small Molecule/RNAi Suppressor Screens Using HD Primary Cultures

We performed dual RNAi and small molecule screens to identify HD toxicity suppressors, and assayed for suppression of Htt138Q aggregate formation, in addition to reversion of mutant Htt138Q morphology towards Htt15Q controls. For RNAi screening, we wanted to identify novel targets for HD therapeutic development, and focused on a kinase/phosphatase RNAi library (468 genes) that would potentially contain targets of high value for chemical inhibition. For small-molecule screening, we tested libraries enriched for FDA-approved drugs, including the NINDS Custom Collection 2, BIOMOL ICCB Known Bioactives Collection and the Prestwick 1 Collection. This allowed screening of ≈2600 approved compounds, potentially facilitating the advancement of screen hits to clinical trials. For compound screening, we verified that addition of 0.2% DMSO to primary cultures does not significantly alter neuronal morphology or Htt138Q aggregation characteristics (Table 1). Known suppressors of Htt polyQ aggregation, including *C2-8*, *GW5074*, *Juglone*, *Radicicol*, and *Rapamycin*, were tested for efficacy in our assay [24]–[28]. Although all control compounds reduced Htt138Q aggregation, none reverted the morphology profiles of Htt138Q expressing neurons towards Htt15Q controls (Table 1). Instead, these compounds caused reduced axon outgrowth, neuromere size, and suppressed GFP expression over a wide concentration range, suggesting these compounds have neurotoxic properties.

**Table 1:** Effect of positive control compounds on Htt138Q aggregate formation and neurite morphology (p-values listed) in *Drosophila* primary neural culture system.

Compound	CAS#	Well Conc. (mM)	Htt Culture	Mutant Htt 138Q aggregation*	Mutant Htt138Q morphology rescue**	n =
C2-8	300670-16-0	2	138Q	***0.0072745***	0	6
C2-8		0.4	138Q	0.216406	0	6
C2-8		0.08	138Q	0.0679971	0	6
C2-8		0.016	138Q	0.381982	0	6
C2-8		0.0032	138Q	0.436453	0	6
GW5074	220904-83-6	2	138Q	***0.000242646***	0	6
GW5074		0.4	138Q	0.414394	0	6
GW5074		0.08	138Q	0.859258	0	6
GW5074		0.016	138Q	0.193046	0	6
GW5074		0.0032	138Q	0.062019	2.54 E-14	6
Juglone	481-39-0	100	138Q	***0.00062262***	0.00000361	6
Juglone		20	138Q	0.999965	0	6
Juglone		4	138Q	0.999994	0	6
Juglone		0.8	138Q	0.908447	0	6
Juglone		0.16	138Q	0.640557	1.26 E-11	6
Radicicol	12772-57-5	100	138Q	***9.17E-66***	0	6
Radicicol		20	138Q	***0.0000587***	0	6
Radicicol		4	138Q	0.671473	0	6
Radicicol		0.8	138Q	***0.0313196***	0	6
Radicicol		0.16	138Q	***0.0116727***	0	6
Rapamycin	53123-88-9	4	138Q	***0.00161737***	0	6
Rapamycin		0.8	138Q	0.134587	0	6
Rapamycin		0.16	138Q	0.151829	0	6
Rapamycin		0.032	138Q	0.382367	0	6
Rapamycin		0.0064	138Q	0.290667	0	6
DMSO	67-68-5	0.2%	138Q	1	0	360
DMSO	67-68-5	0.2%	15Q	0	1	144

*p<0.05 indicates Htt138Q aggregate formation is inhibited (bold, italics).

**p>0.05 indicates Htt138Q drug-treated culture have morphology similar to Htt15Q control cultures.

**Figure 2:**
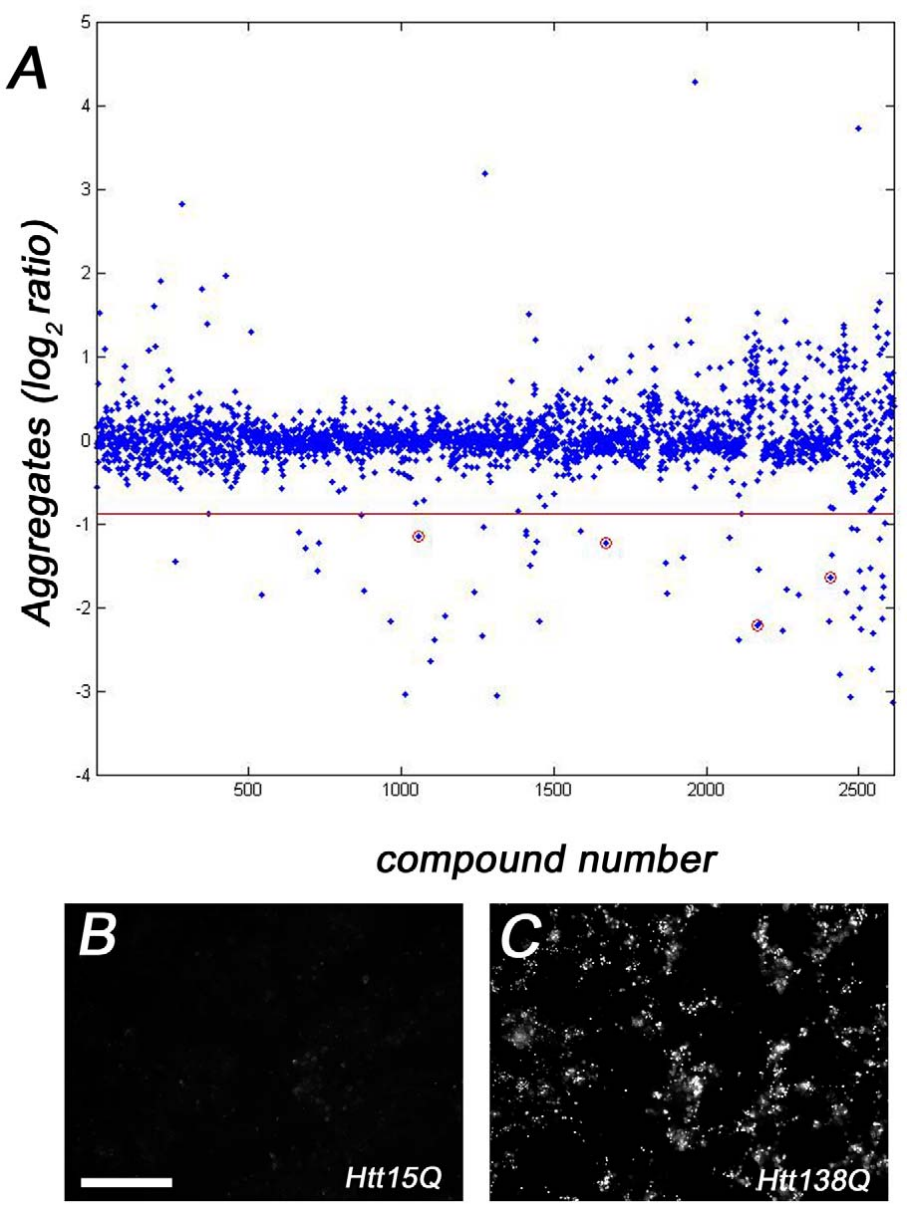
Htt138Q aggregation inhibition screening in primary neural cultures using custom algorithms. (**A**) Scatter plot indicating the extent of Htt138Q aggregation following treatment with ∼2600 small molecules. Log_2_ ratio of Htt138Q aggregates (small molecule treated well/DMSO treated well from the same screen plate) is plotted. The red line denotes two standard deviations from the mean Log_2_ ratio observed in the screen data set. Circled wells correspond to compounds that suppress aggregate formation and were subsequently analyzed in downstream validation studies. (**B,C**) Representative data set images collected via automated microscopy and analyzed with algorithms. (**B**) Htt15Q control cultures have few aggregates, while mutant Htt138Q cultures (**C**) have numerous aggregates. The exposure time used for image collection was optimized for Htt aggregate detection, which has a higher signal intensity than soluble Htt. This avoided pixel saturation at the upper end of the aggregate dynamic range, ensuring accurate aggregate quantification, although soluble Htt is not readily detectable in automated microscopy images. Image analysis was performed as described in the materials and methods. Scale bar: 200 µm.

**Figure 3:**
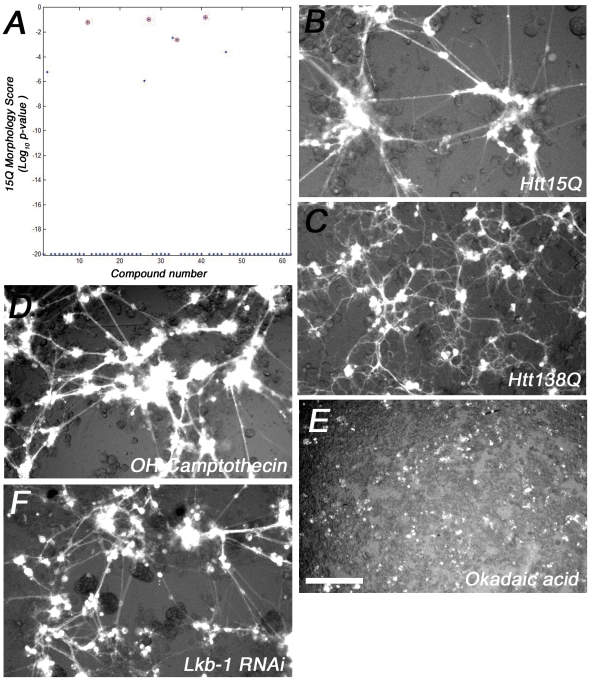
Morphological analysis of Htt138Q aggregation inhibitors. (**A**) P-value scatter plot illustrating the ability of a subset of Htt138Q aggregation inhibitors to revert neuronal morphology towards Htt15Q controls. Circled compounds are the Camptothecin aggregation inhibitors. For morphological analysis, neurite (short, medium, long and average neurite length) and neuromere features (small, medium, large, average neuromere area) were used to compute statistical significance. (**B–E**) Representative automated microscopy images showing the neuronal morphology profiles of *Drosophila* primary neural cultures plated on plastic, optical-bottom, 384-well plates. (**C**) Htt138Q primary neural cultures have dysmorphic neuronal profiles relative to Htt15Q controls (**B**). (**D, F**) Rescue of Htt138Q mutant morphology by treatment with 10-Hydroxy Camptothecin or Lkb-1 knockdown via RNAi. (**E**) An example of a small molecule (Okadaic acid) found to suppress Htt138Q aggregation, but which exacerbates the mutant Htt138Q mutant morphology. Scale bar: 200 µm.

**Figure 4:**
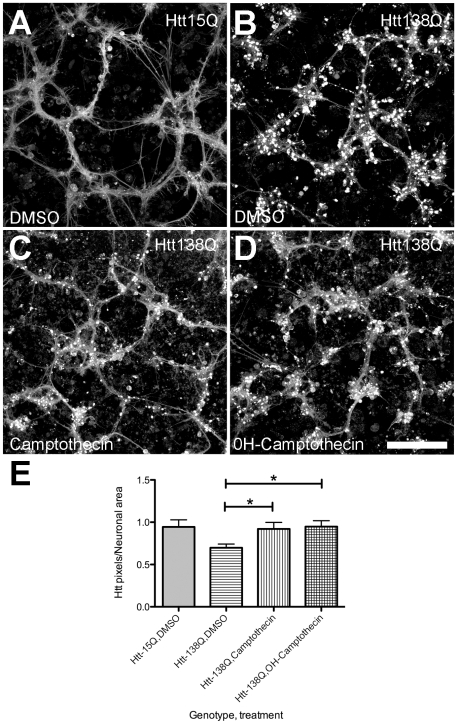
*In vitro* validation of small molecule screen hits. Confocal microscopy images of primary cultures plated on glass coverslips and treated with either DMSO (**A,B**) or test compounds (**C,D**). Primary neural cultures expressing Htt138Q have numerous aggregates in neurite processes and surrounding the cell bodies (**B**), while control Htt15Q expressing cultures do not (**A**). Htt15Q is soluble and fills most neurite processes. Treatment of Htt138Q expressing cultures with Camptothecin (**C**) or 10-OH-Camptothecin (**D**) at 56 µM reduces aggregate formation and increases the proportion of soluble Htt138Q which fills neurite processes. (**E**) Quantification of altered Htt138Q distribution following Camptothecin treatment. An increase in the number of Htt138Q pixels/neuronal area (Htt-RFP pixels/neuromere and neurite GFP pixels) is observed in mutant cultures, suggesting an increase in Htt138Q solubility after drug treatment. * p<0.05, n = 4, Scale bar: 100 µm.

To identify new potential therapeutic compounds that were not neurotoxic, but that were able to suppress mutant Htt toxicity in our model, we conducted RNAi/compound screens in duplicate. All wells were visually scored independently by two investigators to identify agents that either suppressed aggregation, or reverted mutant Htt138Q neural profiles towards Htt15Q controls. From the visual-based screens, three novel suppressors of Htt polyQ toxicity were identified: 1 RNAi hit (*lkb1*), and 2 compounds (Camptothecin and 10-Hydroxycamptothecin). Lkb1 is a known tumor suppressor and a negative regulator of the mTOR/Insulin pathway, which has important roles in autophagy and nutrient sensing [29], [30], while the Camptothecins function as DNA Topoisomerase 1 (*Top1*) inhibitors [31]. In addition to visual inspection of the screen plates, automated microscopy was used to record images of the compound-treated plates for subsequent morphometric analysis using custom algorithms. For automated microscopy, three images per well were taken at different sites for each channel (GFP/RFP) to facilitate hit identification and increase statistical power. Htt138Q aggregation was first quantified, leading to the identification of 62 compounds that significantly suppressed Htt aggregate formation (Fig. 2, Table S1). Subsequently, wells where aggregate formation was inhibited were re-evaluated to determine if any treatments were able to revert the mutant Htt138Q morphology profiles towards that of Htt15Q controls. Of the 62 compounds that were found to inhibit Htt138Q aggregate formation, 8 compounds were identified that improved the Htt138Q induced morphological defects (Fig. 3A, Table 2). Unmasking of the identities of the 8 compounds revealed that 4 compounds were Camptothecins, in agreement with the visual scoring observations. In addition, two Na+/K+ ATPase inhibitors, and a Glutathione-S-Transferase inhibitor were also identified as being capable of suppressing aggregate formation and rescuing the mutant Htt138Q culture morphologies towards the control state (Table 2). We subsequently validated screen hits, focusing on the targets that overlapped in both the visual scoring and morphometric computational analysis. The Lkb-1 target was validated with three independent dsRNAs (amplicons DRSC16481, DRSC36925, DRSC36926). Each amplicon improved Htt138Q mutant morphology with statistical significance, but did not inhibit aggregate formation (Table 3). The Camptothecin and 10-Hydroxy-camptothecin small molecules are structural analogues, and were found to reduce aggregate formation in addition to partially reverting dystrophic morphology profiles over a range of concentrations (56 µM, 5.6 µM) (Fig. 4). These compounds alter Htt138Q localization within neurons, such that it more closely resembles the distribution of Htt15Q in control cultures (Fig. 4).

**Table 2:** Compounds found to inhibit mutant Htt138Q aggregation and revert primary culture morphology towards Htt15Q controls*.

I.D.	Compound	CAS#	Function	Htt Culture	Htt138Q Aggregate Log_2_ ratio	Aggregate Suppression Significance** (p-value)	Morphology Statistical Significance*** (p-value)	Library	ICCB Plate I.D., Well #	Well Conc
**1**	**Camptothecin**	7689-03-4	topoisomerase I inhibitor, antineoplastic	138Q	−1.637238	0.00000365	0.151249	BIOMOL2	1791, N06	26 uM
**2**	**Camptothecin**	7689-03-4	topoisomerase I inhibitor, antineoplastic	138Q	−1.22608	0.00000927	0.109087	Prestwick	1568, H21	10 uM
**3**	**Camptothecin**	7689-03-4	topoisomerase I inhibitor, antineoplastic	138Q	−1.152003	0.00000000	0.06008	NINDS	1921, N08	20 uM
**4**	Etoposide	33419-42-0	topoisomerase II inhibitor, antineoplastic	138Q	−0.881592	0.00003212	0.003217	Prestwick	1569, O13	6.8 uM
**5**	**10-OH-Camptothecin**	64439-81-2	topoisomerase 1 inhibitor, antineoplastic	138Q	−2.21028	0.00000012	0.00242	BIOMOL2	1791, B06	26 uM
**6**	Ouabain	630-60-4	Na+/K+-ATPase inhibitor	138Q	−1.050767	0.00000268	0.000246	BIOMOL2	1792, B13	17 uM
**7**	Proscillaridin A	466-06-8	Na+/K+-ATPase inhibitor	138Q	−0.876806	0.00020794	0.000006	Prestwick	1571, E13	7.5 uM
**8**	Ethacrynic acid	58-54-8	GST inhibitor	138Q	−1.088665	0.00003454	0.000001	Prestwick	1568, D20	13.2 uM

*Compounds listed correspond to hits shown in Figure 3A. Bolded compounds are the circled hits.

**P<0.05 indicates that Htt138Q aggregate formation is inhibited.

***P>0.05 indicate mutant Htt138Q neurite morphology is reverted towards Htt15Q controls.

**Table 3:** RNAi validation. Effect of Lkb-1 or Top knockdown on Htt138Q aggregate formation and rescue of mutant culture morphology.

Gene Target	dsRNA amplicon	Off-targets	Htt Culture	Aggregate Suppression Significance (p-value)*	Morphology Statistical Significance (p-value)**	n =
LKB-1	DRSC16481	0	138Q	0.995894	0.152692	12
LKB-1	DRSC36925	0	138Q	0.730001	0.979141	12
LKB-1	DRSC36926	0	138Q	0.89236	0.702101	12
Top 1	DRSC36056	0	138Q	0.833703	0.428127	12
Top 1	DRSC20295	0	138Q	0.968551	0.015374	12
Top 2	DRSC36057	0	138Q	0.993983	0.208051	12
Top 2	DRSC03459	0	138Q	0.960684	0.35244	12
Top 3α	DRSC03460	0	138Q	0.980167	0.102255	12
Top 3α	DRSC37672	0	138Q	1	0	12
Top 3β	DRSC18724	0	138Q	0.288516	0.331471	12
Mock	N/A	N/A	138Q	1	0	12
Mock	N/A	N/A	15Q	0	1	72

*Htt Aggregates. P<0.05 Indicates suppression of Htt138Q aggregate formation.

**P>0.05 indicates mutant Htt138Q neurite morphology is reverted towards Htt15Q controls.

### Genetic and Pharmacological Validation of Screen Hits

**Figure 5:**
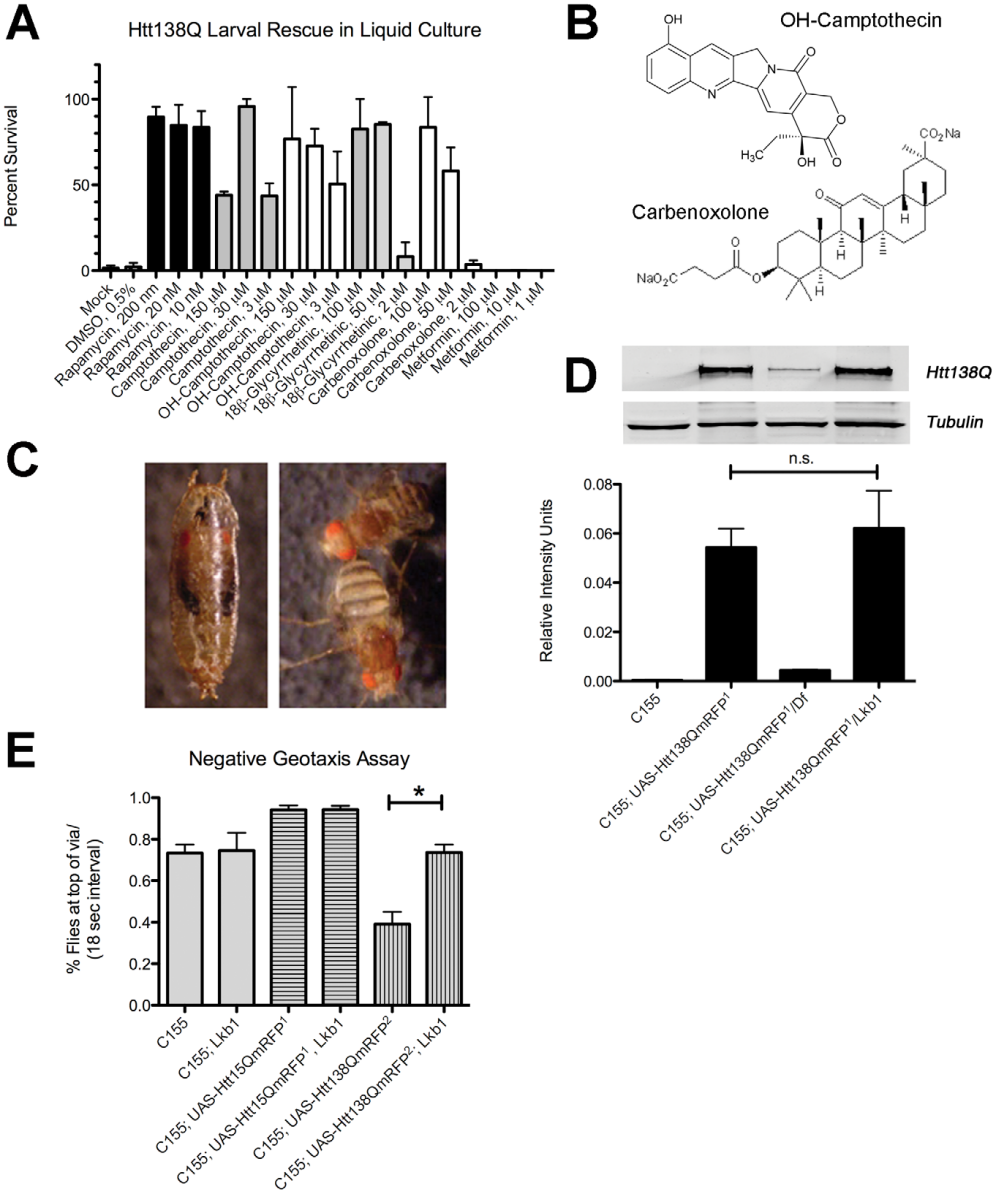
*In vivo* validation of screen hits. (**A**) Survival frequency scores for HD larvae (*Elav^c155^-GAL4*; *UAS-Htt138Q^2^/+*) after 5-day drug dosing in liquid culture. (**B**) Chemical structures of the Camptothecin and 18β-Glycyrrhetinic acid class of small molecules found to rescue Htt138Q toxicity *in vivo*. (**C–E**) Genetic interaction studies to assess the effect of *lkb1* kinase reduction on Htt138Q toxicity. (**C**) Pan-neuronal expression of Htt138Q^1^ causes pupal lethality (left) which can be rescued with the introduction of an *lkb1* heterozygous background. (**D**) Quantitative Western blot analysis demonstrating *lkb1*-rescued HD adults have normal Htt138Q expression levels. A control deficiency, *Df(3L)vin*, which reduces Htt138Q expression is shown for comparison. (**E**) *Lkb1* mutation rescues the climbing behavior of HD flies. 25 day-old Htt138Q flies (*C155;UAS-Htt138QmRFP^2^*) have impaired climbing behavior as compared to controls. Introduction of an *Lkb1^4A4-2^* trans-heterozygous mutation into the *Htt138Q^2^* background improves climbing ability. * p<0.05.

To further examine the RNAi/small molecule screen hits, we assayed *in vivo* efficacy by testing their ability to rescue lethality in our *Drosophila* HD model. Htt138Q expression in the nervous system results in late stage pupal lethality when animals are reared on standard media. Animals undergo metamorphosis but fail to eclose. In liquid culture, the longevity of the Htt138Q expressing animals is reduced and larvae perish during the 2^nd^ instar stage, likely due to drowning from decreased motility. Rapamycin, a well characterized mTOR inhibitor [25], suppresses neurodegeneration in various HD models, and we found it enhanced viability of Htt138Q expressing larvae reared in liquid culture in a dose-dependant fashion compared to DMSO-treated controls (Fig. 5A). Using this assay, we found that Camptothecin and 10-Hydroxycamptothecin also increased larval longevity *in vivo*, but to a lesser extent than Rapamycin (Fig. 5A). 10-Hydroxycamptothecin is more efficacious than Camptothecin, possibly due to solubility differences since Camptothecin readily precipitates when added to cultures. Specific inhibitors of Lkb1 were not available for *in vivo* testing. Given its role as an upstream regulatory kinase of the mTOR/Insulin pathway, we tested additional pharmacological agents that regulate this pathway, including Metformin (an mTOR pathway activator and oral anti-diabetic drug) [32], [33] and 18β-Glycyrrhetinic acid (a putative mTOR inhibitor that acts through PI3K/AKT) [34], [35]. Metformin could not revert Htt138Q-incuded lethality. However, 18β-Glycyrrhetinic acid was almost as efficacious as Rapamycin (Fig. 5A). We also tested an analogue of 18β-Glycyrrhetinic acid that has increased solubility: Carbenoxolone, and found it to have comparable activity. 18β-Glycyrrhetinic acid is non-toxic and has been used as a commercial sweetener, making it an attractive candidate for future studies for suppressive effects in mammalian HD models. Rapamycin, in contrast, has numerous toxic side-effects that can impact kidney and lung function, cause increased risk of infection, and lead to hyperlipidemia [36]–[40].

To further examine the role of the Lkb1/Insulin pathway in the suppression of HD toxicity, we conducted genetic interaction studies with *lkb1* loss-of-function mutations. While pan-neuronal expression of Htt138Q^1^ causes pupal lethality, the introduction of a heterozygous *lkb1* null mutation into the Htt138Q background suppresses lethality. We observed no C155/+; UAS-Htt138Q/+ adult escapers at 25°C (n = 83 pupae), however, the introduction of an *Lkb1^4B1-11^/+* or *Lkb1^4A4-2^/+* allele into this background led to an adult escaper frequency of 1.8% (n = 110 pupae) and 3.7% (n = 81 pupae) respectively. Using quantitative Western blot analysis, we found that the introduction of *lkb1* trans-heterozygous alleles does not reduce Htt138Q protein levels (Fig. 5D), suggesting the suppressive effects are not tied to altering Htt expression. This is in contrast to another rescuing deficiency we identified in an independent screen, *Df(3L)vin7*, which significantly decreases Htt138Q expression and yields an escaper frequency of 25.9% (n = 85 pupae). The *Lkb1* heterozygous animals expressing Htt138Q are viable and have relatively normal walking ability, although they do not inflate their wings (Fig. 5C, Movie S1). To further investigate the relationship between *lkb1* and mutant Htt138Q toxicity, we introduced the *Lkb1^4A4-2^/+* allele into a weaker Htt138Q expressing strain (C155/+; UAS-Htt138QmRFP^2^/+) which is adult viable so that we could evaluate climbing behavior as an indicator of motor performance. From negative geotaxis assays performed on 25-day old flies, we found that introduction of an *Lkb1^4A4-2^/+* mutant background enhanced performance only in the C155:Htt138Q background, but had no effect on either C155 or C155:Htt15Q control backgrounds (Fig. 5E). This suggests that the toxicity effects of Htt138Q in neurons, is associated with Lkb-1 signaling. Since RNAi knockdown, as performed in our primary culture screening assay, is representative of a hypomorphic situation, and the *in vivo lkb1* rescue studies we conducted were haplo-insufficient, partial knockdown screening can be advantageous to uncover therapeutic targets. Full knockdown of *lkb1* would not have revealed beneficial effects, as homozygous *lkb1* null mutants are lethal and have cell polarity defects [41].

To investigate the mechanism of action of Camptothecins in suppression of Htt138Q neurotoxicity, we performed genetic loss-of-function studies with target effector proteins. Since Camptothecins function as *Top1* inhibitors, we reasoned that *Top1* RNAi knockdown in primary cultures should phenocopy Camptothecin treatment and suppress HD pathology. RNAi knockdown of *Top1* or other annotated *Drosophila Top* genes (*Top2, 3α or 3β*), either singularly or in combination, did not suppress Htt aggregation (Table 3). Knockdown of the Tops did, however, partially revert the mutant Htt138Q neurite morphology towards controls. To extend these studies *in vivo* we introduced a heterozygous *Top1* null allele into the HD model background, but this had no effect on Htt138Q-induced pupal lethality, as no adult escapers were observed. Given that Camptothecins have a robust effect on Htt138Q aggregation inhibition, while Top-knockdown does not, these results suggest that Camptothecins may act through a Top1-independent pathway to suppress Htt138Q aggregation.

The compounds that suppress Htt138Q aggregate formation, and/or rescue neurite morphology in our system, can be grouped into two classes based on their structures (Figure S1). The Camptothecins, GW5074, Radicicol, and Etoposide form one class that have partially overlapping backbone ring-structures, while 18β-Glycyrrhetinic acid, Ouabain and Proscillaridin-A form a second class that share a similar steroid-like backbone. It will be interesting to conduct further structure-function analysis to determine a minimal architecture that is required for these compounds to rescue Htt138Q-induced pathology.

## Discussion

We have used *Drosophila* primary neural cultures isolated from an HD model to screen for RNAi and small molecule suppressors of expanded polyQ Htt-induced toxicity. Cultures expressing a 588 amino acid fragment of human Htt containing an expanded polyQ domain (138Q) display robust cytoplasmic Htt aggregates, and have dystrophic neurites compared to Htt15Q control cultures. To identify supressors of expanded polyQ Htt toxicity, we screened for compounds and RNAi targets that reduce aggregate formation and revert morphological defects towards the control state. Our screening resulted in the identification of *lkb1*, an upstream kinase regulator of the mTOR/Insulin pathway, as a suppressor of mutant Htt toxicity. We also identified multiple compounds that have promising HD therapeutic efficacy: 18β-Glycyrrhetinic acid, Carbenoxolone, and the Camptothecins. Due to the efficiency of conducting screens in cell culture, and the increased physiological relevance of primary neurons, this methodology represents a powerful approach to identify modifiers for other neurodegenerative disorders.

LKB-1 knockdown was found to suppress mutant Htt toxicity in our system, as it rescued the dysmorphic primary neural culture morphology *in vitro* and restored viability *in vivo*. LKB-1 has been extensively studied, and mutations in the locus result in the Peutz Jeghers Syndrome (PJS) [42], [43]. In *Drosophila*, loss of LKB-1 in the embryonic nervous system blocks apoptosis and results in hyperplasia [44]. How a partial LKB-1 knockdown elicits its beneficial effect in our system is still uncertain, although decreased levels of LKB-1 may reduce apoptosis caused by mutant Htt. LKB-1 lies upstream of many pathways that have previously been implicated in HD, including the mTOR/autophagy pathway [25], [45], [46] and the Insulin/AMPK signaling network [47], [48]. Recently our findings were corroborated in vertebrates as activation of AMPK, the main kinase target of LKB-1, was found to potentiate striatal neurodegeneration in HD [49].

Several compounds that suppressed mutant Htt toxicity in our primary culture system have previously been shown to have neuroprotective effects in mammalian systems, indicating that the assay with *Drosophila* primary cultured neurons has translational capacity. Compound GW5074 inhibited mutant Htt aggregate formation in our system, and also reduced striatal degeneration in the NP-3 mouse HD model [28]. Similarly, 18β-Glycyrrhetinic acid, which rescued HD toxicity *in vivo* in our *Drosophila* assays, has been shown to suppresses neurotoxicity in a PC12 cellular stress model [34].

By analyzing the molecular structures and mechanisms of action of the small molecules identified as mutant Htt suppressors, new avenues to investigate the biology of HD pathogenesis have been uncovered. 18β-Glycyrrhetinic acid, and Carbenoxolone, which were used to pharmacologically manipulate LKB-1 dependent pathways and rescue HD toxicity *in vivo*, have also been reported to block gap junction activity [50]. Although this mechanism was not further investigated, it is an intriguing approach for future characterization in HD pathology. Recently there have been several reports that mutant Htt expressed in glia can trigger neuronal defects [7], [51]–[53]. In addition, postmortem analysis of HD patient brain samples revealed increased activated astrocytes and reactive microglia in the striatum and cortex compared to similar aged non-diseased brains [54]. Gap junctions allow astrocytes to communicate via elaborate networks, and there is evidence that cell death signals can be propagated through gap junction networks [55]. Therefore, modulating gap junction activity with non-toxic compounds such as 18β-Glycyrrhetinic acid or Carbenoxolone might have neuroprotective benefits. 18β-Glycyrrhetinic acid derivatives are particularly interesting because they have already been evaluated in two clinical trials for other indications (ClinicalTrials.gov Identifier: NCT00384384 and NCT00759525), and are widely used as commercial sweeteners. Recently, an 18β-Glycyrrhetinic acid derivative was found to be efficacious in the treatment of two mouse models of Amyotrophic Lateral Scleosis (ALS) and an Alzheimer's Disease model, further supporting the potential therapeutic value of this class of compounds for neurodegenerative diseases [56].

Camptothecins were very effective at suppressing the dystrophic neuronal profiles and mutant Htt aggregation in our assay. Camptothecins are potent anti-cancer drugs that block cell division through several mechanisms including the introduction of DNA replication-dependant double-stranded breaks which trigger apoptosis, and down regulation of Top-1 by activation of proteasome pathways. In quiescent neurons, Camptothecins most likely cause transcriptional repression as a result of collisions between RNA polymerase and immobilized Top-1/Camptothecin complexes linked to the DNA. In our system, the benefit of Camptothecin treatment could theoretically be related to decreased Htt transgene expression, although we did not observe any decrease in Htt-mRFP fluorescence, even after one week of continuous exposure to the drug. Since targeted knockdown of mutant Htt via siRNA has been found to be effective at reversing disease progression in mouse models, small molecule transcriptional repressors may offer another therapeutic avenue to control HD [57]. Although toxicity issues have been reported in neural cultures following Camptothecin treatment, we did not observe morphological defects in our *Drosophila* HD model [58]. Camptothecins have been reported to regulate a number of different pathways, including activation of the ubiquitin/proteasome system and upregulation of mitochondrial biogenesis. These secondary Camptothecin effects could alleviate toxic Htt cellular stress by removing toxic Htt species or restoring energy homeostatis [59]–[62].

There has been debate in field about the contribution of Htt aggregates to disease pathology for many years. Several studies have shown that Htt aggregates accumulate in fine neuronal processes such as axons and dendrites, and block axon-transport to negatively impact cell heath [17], [63]–[65]. Real-time imaging experiments have suggested that soluble Htt, and not aggregates, correlate better with cellular toxicity [66]. Given this controversy, we chose not to use aggregate suppression as the sole metric to identify small molecules and RNAi knockdown probes that have therapeutic value and included an additional parameter: neurite morphology. We found that neurite processes are sensitive to mutant polyQ-expanded Htt and offer a means of identifying drugs and RNAi knock-downs that have non-specific toxicity effects. Using this assay we were able to identify compounds and RNAi knockdowns that have potential therapeutic value. Although our studies cannot conclusively demonstrate a physiological link between aggregate inhibition and improved neuronal health, we did discover compounds that improved neurite morphology in addition to reducing mutant Htt aggregation, providing anecdotal evidence that in some cases aggregates may have toxic properties.

*Drosophila* models of neurodegenerative disease have been a powerful tool for understanding mechanisms of neurodegeneration for more than a decade, and have more recently been applied directly to drug discovery as well [67]–[74]. Aside from genetic tools in *Drosophila* and the host of neurodegenerative disease models available, it is an attractive model for conducting suppressor screens given the lack of gene redundancy often observed in mammals. While single gene knock-down studies often fail to produce robust phenotypes in mammals, this is not the case in *Drosophila* [75], [76]. We have found that the complex neural morphologies of *Drosophila* primary cultures can also provide sensitive information about the general cell physiological status of a disease model. The algorithms that we have used in this study can help quantify complex morphologies can also facilitate the identification of disease modifying genes [23]. Live imaging, as presented here, has the advantage over traditional cell staining experiments in that the fine neurite morphology of cultures is preserved. Detergents and washes needed for immunofluorescence-based assays can disrupt fine cellular processes and introduce artifacts, which reduce assay sensitivity and introduce noise. Live-cell imaging also makes it possible to collect different time points in a single experiment, which not only reduces labor but also enables one to track the effect of a compound or gene knockdown over time. Because of the ease and speed of conducting RNAi and compound screens in *Drosophila* primary culture systems, this methodology offers an attractive approach to identify disease-modifying agents for neurodegenerative diseases.

## Methods

### Primary Cell Culture

*Elav^c155^-GAL4* virgins were collected en masse and crossed to either *UAS-Htt138QmRFP^1^,UAS-mCD8-GFP* or *UAS-Htt15QmRFP^1^,UAS-mCD8GFP* males to generate embryos for primary culture preparation. Neuroblasts were isolated as previously described [22].

### Western Blotting

Embyronic lysates (n = 4/genotype) were prepared from control and Htt expressing strains (50 mM Tris, pH 8.0, 150 mM NaCl, 0.1% SDS, 1.0% NP-40 (IgePal), 0.1% sodium deoxycholate plus protease inhibitors (cOmplete-mini, Roche)), and protein content was quantified using a BCA kit (Pierce). Protein samples (10 µg/lane) were analyzed using standard SDS-PAGE/Western blotting techniques, and quantified using an Odyssey Infrared Imaging System (Li-Cor). For immunoblotting, antibodies were used at the following concentrations: mouse anti-Tubulin (6-11B-1, Sigma-Aldrich T7451) at 1/60,000, mouse anti-human Htt (MAb 2166, Chemicon) at 1/1,000, and goat-anti-mouse IR800 secondary (LI-COR 926-32210) at 1/3,000.

### Compound screening

Primary cultures were re-suspended directly in Shields and Sang M3 media (Sigma) supplemented with 10 U/mL penicillin, 10 µg/mL streptomycin, 200 ng/mL insulin, and 5% fetal bovine serum. 100 nL of compounds from arrayed small-molecule libraries (NINDS Custom Collection 2, Prestwick1 Collection, BIOMOL2 ICCB-Longwood Known Bioactives High Concentration, various concentration from 1–15 mM in DMSO) were applied to 50 µL of cultures 24 hours after plating on optical bottom 384-well plates (Corning 3712). The neuroblast density was 18,500 cells/well. The primary screen was carried out in duplicate and hits were validated with 12 additional replicate wells.

### RNAi Screening

dsRNAs (250 ng/well) were aliquoted onto microscopy plates and then 10 µL of neuroblasts were applied to a density of 18,500 cells/well. Cultures were incubated for 3 days with dsRNAs to achieve gene knockdown. Shields and Sang M3 media (Sigma) supplemented with 10 U/mL penicillin, 10 µg/mL streptomycin, 200 ng/mL insulin, and 5% fetal bovine serum was then added to cultures to bring assay volume to 50 µL. The *Drosophila* RNAi Screening Center (DRSC) whole genome kinase/phosphatase library (468 genes, 3 amplicons/gene) was screened in duplicate, and hits were validated using additional dsRNA amplicons containing no off-targets. For RNAi validation studies, dsRNAs were synthesized from T7-tailed DNA templates using the MEGAshortscript T7 transcription kit (Ambion). Synthesized dsRNAs were purified with RNeasy kits (Qiagen) before use in cell culture experiments. The T7-tailed oligonucleotides used to generate DNA templates from w^1118^ genomic DNA are as follows: Lkb-1: DRSC16481 (GCCGTCAAGATCCTGACTA/CTCCGCTGGACCAGATG), DRSC36925 (GCAACTCCACGGTGATACCT/ATGCAGGACGTCAGCTTCTT), DRSC36926 (ATTGCGGCGAACTTACTTTG/TAATCCTCACCAGGCACACA); Top-1: DRSC36056 (GAGAATGTGCAGGGACAGGT/GTCGATGAAGTAAAGGGCCA), DRSC20295 (GGAGGAGGAGAAGCGTG/GCGCCGCTTGATCATG); Top2: DRSC36057 (CACAGCGACAGAAGCATCAT/TTCTTGTATTCCCTCGTGGC), DRSC3459 (TTTGCCAGAGCGATATCTC/CCATAGTGGCTCGATCTTTT); Top3α: DRSC3460 (TTAAACGTGGCTGAGAAGAA/GCCCACGCCCTTTTTCA), DRSC37672(GTGGTCCTGACCGAACAGAT/AGGTTTTGTACCAACCGCTG); Top3β: DRSC18724(GCGGACTTCGGTGAGGA/CGCTGGCAGATGTTGTTG).

### Microscopy

For high-content screening, mature 7-day old cultures were imaged with an ImageXpress^MICRO^ robotic microscope (Molecular Devices, Sunnyvale, CA) using a 10× objective and FITC/Cy3 filter sets. Images were 1392×1040 pixels, or 897×670 micrometers. Laser-based autofocusing was used to locate plate bottoms, and then image-based focusing was used to resolve fluorescently labeled neurons over a 48 µm range. The GFP and mRFP channels were imaged in the same focal plane, with exposure times of 850 and 400 ms respectively. Three sites were imaged per well for each treatment group, and the screen was done in duplicate. For confocal microscopy of primary cultures, neuroblsts were plated on poly-L-lysine coated chambered cover slips (LabTekII, 0.8 cm^2^/well) at 18,300 cells/well in 50 µL volume. Small molecules were added to cultures 24 hours after plating, incubated for 7 days, and then imaged with a Leica TCS-SP2 confocal LSM microscope.

### Digital Image Analysis of High Content Screening Data Sets

Neuronal morphological analysis and Htt aggregate quantification for automated microscopy images was performed as previously described [23], [77]. In brief, Htt138Q aggregates were quantified as the total number of pixels/image with intensity higher than an empirically set threshold. To quantify neuronal morphologies, cell body clusters (neuromeres) and neurites were extracted from images using our custom algorithms [21]. The *log*_2_ transformed areas of cell body clusters were found to fit a Gaussian mixture model (GMM) and therefore were separated into three bins (small, medium, and large). Absolute counts of neuromeres/bin were tabulated for all images. Neurite segment lengths were similarly clustered into three groups (short, medium, and long) using the *K*-means method and then quantified. Cell cluster and neurite counts were converted into percentages to control for any variation in cell number between wells arising from pipetting error. Mean neuromere area and neurite length for each image were also calculated to give a total of eight morphological metrics for image morphology quantification. For statistical analysis, *p*-values for each morphological feature were calculated using a two sample *t*-test (e.g. small neuromere feature of Htt138Q drug-treated cultures *versus* small neuromere feature of Htt15Q DMSO cultures). The resultant morphological *p*-values for individual features were then integrated into a single *p*-value using the Fisher method [78] defined by the equation 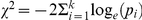, where *k* represents independent tests, *p_i_* is the *p*-value of the *i*-th feature. The combined statistic has a *χ* ^2^ distribution with 2*k* degrees of freedom under the joint null hypothesis. This method works well in cases where the evidence against the null-hypothesis is spread across different features. Excluded from analysis were compound-treated wells with <6 images, out-of-focus images, or images that lacked cell profiles altogether.

### *In vivo* Rescue studies

For small molecule *in vivo* rescue studies, *Elav^c155^*; *UAS-Htt138QmRFP^1^,UAS-mCD8-GFP*/+ 1^st^ instar larvae were collected en-masse and dispensed into liquid media (10 larvae/well) containing different concentrations of test compounds (n = 8 replicas/concentration). The cultures were reared at 21°C for 5 days, and the mean number or living Htt138Q larvae (i.e. GFP^+^, Htt138QmRFP^+^, and mobile) was tabulated, and expressed as a percentage of total larvae/well. For genetic rescue studies with *lkb1*, two Htt138Q strains were utilized: a strong expressing line, *UAS-Htt138QmRFP^1^* which is pharate lethal when crossed to *Elav^c155^*, and a weaker expressing line, *UAS-Htt138QmRFP^2^* which survives to adulthood and is viable for a number of weeks. Using the strong line, pharate lethality at 25°C was calculated after *lkb1* (*lkb1^4B1-11^* and *lkb1^4A4-2^* alleles) was introduced into an Elav^c155^,UAS-Htt/+ background. *Lkb1^4B1-11^* is a premature truncation allele (Q98>Stop), and *lkb1^4A4-2^* is an EMS null allele (589 b.p. deletion removing 150 b.p. of the 5′ UTR, the start codon and the beginning of the open reading frame). For Top1 *in vivo* analysis, Elav^c155^,*Top^112^* recombinants were generated and crossed to *UAS-Htt138QmRFP^1^*.

### Negative Geotaxis Assay

*Lkb1^4A4-2^* was crossed into the Elav^c155^, *UAS-Htt138QmRFP^2^* which is adult viable and has weaker Htt138Q expression. Virgin female *Drosophila* were collected and flipped onto fresh media two times per week until the start of the assay. 25 day-old flies (10–15 flies/vial, 4 vials/genotype) were gently tapped to the base of vials, and climbing behavior was video-recorded for 18 seconds (trial). The percentage of flies to reached the top of a vial was tabulated and averaged after 4 trails. Vials were back-lit with a light box to enhance the resolution of the fly climbing trajectories. Statistical analysis was performed using a t-test.

## Supporting Information

Figure S1 Selective compounds tested for their ability to suppress *Htt138Q* neuronal toxicity.
(TIF)

Table S1 Compounds found to inhibit Htt138Q aggregate formation in *Drosophila* primary neural culture screen.
(DOC)

Movie S1 *Elav^c155^-GAL4*;*UAS-Htt138QmRFP^1^*/*lkb1^4A4-2^* rescued adult *Drosophila*. While *Elav^c155^-GAL4*;*UAS-Htt138QmRFP^1^/+* adults are pharate lethal, introduction of a heterozygous *lkb1^4A4-2^* allele suppresses Htt138Q toxicity and results in the emergence of viable escapers. Escapers have normal walking ability, but are unable to unfurl and inflate their wings.
(MPG)
