# Supplementary figures and images for: High-Content Chemical and RNAi Screens for Suppressors of Neurotoxicity in a Huntington's Disease Model
Source: PLoS One. 2011 Aug 31;6(8):e23841. doi: 10.1371/journal.pone.0023841 (PMC3166080; doi:10.1371/journal.pone.0023841)

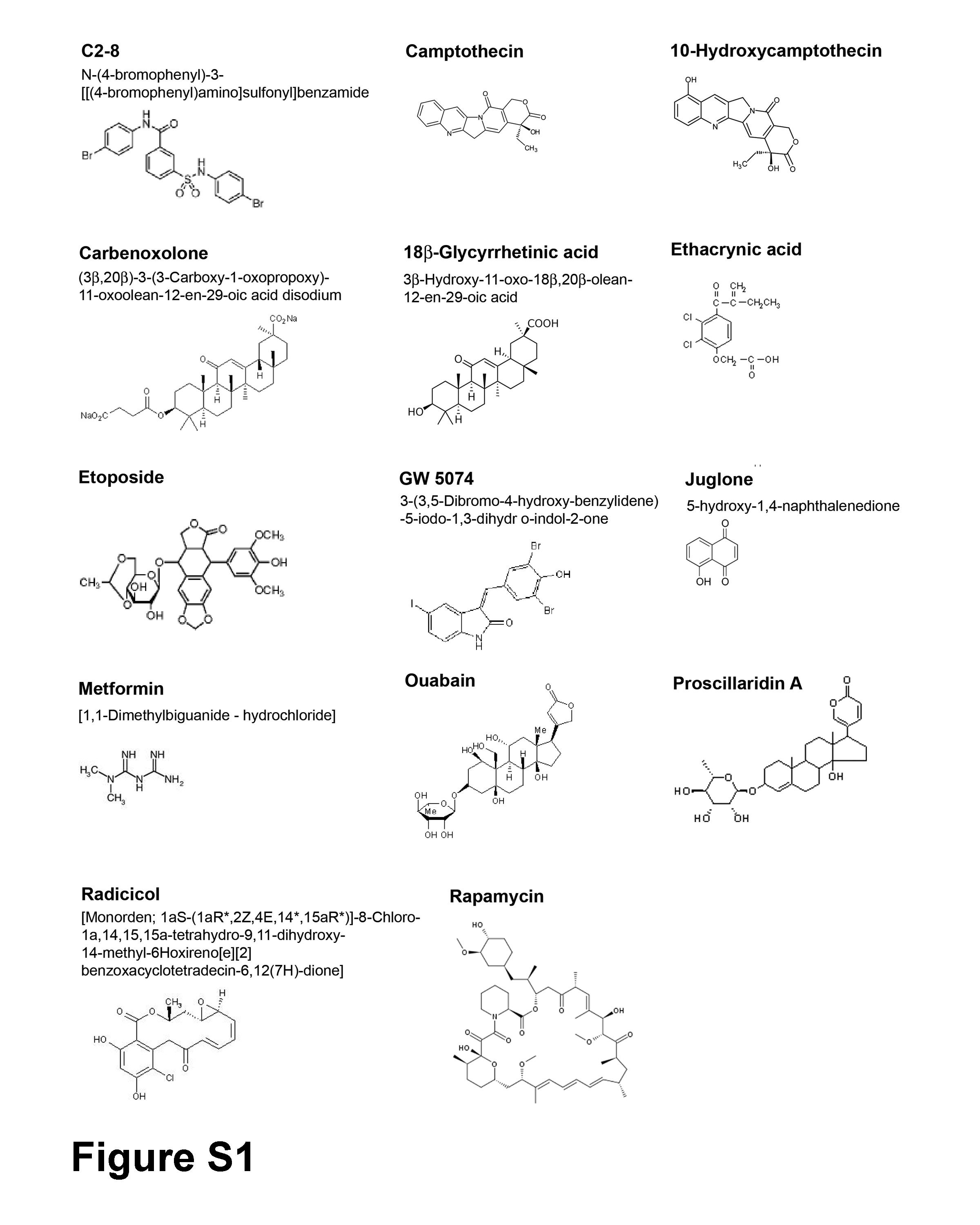

Supplement: Figure S1 — Selective compounds tested for their ability to suppress Htt138Q neuronal toxicity. (TIF) [file pone.0023841.s001.tif]
