# Supplementary material for: High-Content Chemical and RNAi Screens for Suppressors of Neurotoxicity in a Huntington's Disease Model
Source: PLoS One. 2011 Aug 31;6(8):e23841. doi: 10.1371/journal.pone.0023841 (PMC3166080; doi:10.1371/journal.pone.0023841)
Supplement: Table S1 — Compounds found to inhibit Htt138Q aggregate formation in Drosophila primary neural culture screen. (DOC) [file pone.0023841.s002.doc]

**Table S1:** Compounds that inhibited Htt138Q aggregate formation in *Drosophila* primary neural culture screen.

| **I.D.** | **Library** | **ICCB plate** | **Well** | **Library Conc** | CAS# | Compound* | **Function** | **Aggregate Log_2_ ratio** | **Morphological p-value** |
| --- | --- | --- | --- | --- | --- | --- | --- | --- | --- |
| 1 | BIOMOL2 | 1792 | P17 | 5mg/mL | G237 | Cerulenin | Fatty acid biosynthesis inhibitor | -3.139483 | 0 |
| 2 | BIOMOL2 | 1792 | B07 | 0.5mg/mL | EI181 | Okadaic acid | PP1 PP2A inhibitor | -3.077206 | 0 |
| 3 | NINDS | 1922 | K06 | 10 mM | 22862-76-6 | Anisomycin | antiprotozoal, antifungal, protein synthesis inhibitor | -3.055318 | 0 |
| 4 | NINDS | 1921 | L04 | 10 mM | 66-81-9 | Cycloheximide | protein synthesis inhibitor | -3.0364 | 0 |
| 5 | BIOMOL2 | 1791 | O18 | 5mg/mL | CA-100 | A-23187 | Calcium ionophore | -2.809242 | 0 |
| 6 | BIOMOL2 | 1792 | I13 | 5mg/mL | CA-201 | Ionomycin | Ca++ ionophore | -2.735893 | 0 |
| 7 | NINDS | 1921 | P06 | 10 mM | 58-27-5 | Menadione | prothrombogenic agent | -2.64595 | 0 |
| 8 | Prestwick | 1569 | N21 | 2 mg/ml | 20554-84-1 | Parthenolide | anti-inflammatory | -2.393522 | 0 |
| 9 | NINDS | 1921 | P18 | 10 mM | 622-78-6 | Benzyl Isothiocyanate | antineoplastic, antibacterial, antifungal | -2.385704 | 0 |
| 10 | NINDS | 1922 | H15 | 10 mM | 123-31-9 | Hydroquinone | antioxidant | -2.341888 | 0 |
| 11 | BIOMOL2 | 1792 | J07 | 5mg/mL | EI-156 | Staurosporine | kinase inhibitor | -2.313346 | 0 |
| 12 | BIOMOL2 | 1791 | F08 | 5mg/mL | GR300 (Biomol) | Actinomycin D | transcription inhibitor | -2.277917 | 0 |
| 13 | BIOMOL2 | 1792 | F03 | 5mg/mL | GR-312 | Puromycin | protein synthesis inhibitor | -2.254039 | 0 |
| 14 | BIOMOL2 | 1791 | B06 | 5mg/mL | GR-316 (Biomol) | **10-Hydroxycamptothecin** | topoisomerase 1 inhibitor, antineoplastic | -2.21028 | 0.00242 |
| 15 | BIOMOL2 | 1791 | B08 | 5mg/mL | GR-308 (Biomol) | Beta-lapachone | topoisomerase 1 inhibitor, antineoplastic | -2.193166 | 0 |
| 16 | NINDS | 1923 | E05 | 10 mM | 2752-65-0 | Gambogic acid | antiinflammatory, cytotoxic, inhibits HeLa cells in vitro; LD50(rat) 88 mg/kg ip | -2.173426 | 0 |
| 17 | NINDS | 1921 | I15 | 10 mM | 1404-88-2 | Tyrothricin | topical antibacterial (topical) | -2.17232 | 0 |
| 18 | BIOMOL2 | 1791 | N03 | 5mg/mL | PI-122 | Tosyl-Phe-CMK (TPCK) | serine protease inhibitor | -2.166435 | 0 |
| 19 | BIOMOL2 | 1792 | M09 | 5mg/mL | EI-293 | 5-iodotubercidin | ERK-2 inhibitor | -2.139152 | 0 |
| 20 | BIOMOL2 | 1792 | C09 | 5mg/mL | CN-240 | Diphenyleneiodonium Cl | flavoprotein inhibitor | -2.113107 | 0 |
| 21 | NINDS | 1922 | B15 | 10 mM | 29767-20-2 | Teniposide | topoisomerase II inhibitor, antineoplastic | -2.103361 | 0 |
| 22 | BIOMOL2 | 1792 | E11 | 5mg/mL | CM120 | FCCP | mitochondrial uncoupler | -2.014179 | 0 |
| 23 | BIOMOL2 | 1792 | M07 | 5mg/mL | CN-200 | LY-83583 | inhibits NO-activation of guanylate cyclase | -1.879978 | 0 |
| 24 | BIOMOL2 | 1791 | H22 | 5mg/mL | EI-175 (Biomol) | Aristolochic acid | phospholipase A2 inhibitor | -1.851602 | 0 |
| 25 | NINDS | 1920 | D07 | 10 mM | 70-30-4 | Hexachlorophene | antiinfective (topical) | -1.844784 | 0 |
| 26 | Prestwick | 1569 | C06 | 2 mg/ml | 22862-76-6 | Anisomycin | antibiotic, activator of p38 and MAP kinases | -1.83072 | 0 |
| 27 | BIOMOL2 | 1791 | P21 | 5mg/mL | CA-421 | Nigericin | induces intracellular acidification | -1.821227 | 0 |
| 28 | NINDS | 1922 | G11 | 10 mM | 522-51-0, 6707-58-0 [dequalinium] | Dequalinium chloride | antiinfectant | -1.811869 | 0 |
| 29 | NINDS | 1921 | E07 | 10 mM | 54-64-8 | Thimerosal | antiinfective, preservative | -1.798877 | 0 |
| 30 | BIOMOL2 | 1791 | F20 | 5mg/mL | EI-258 (Biomol) | AG-879 | NGF receptor inhibitor | -1.786601 | 0 |
| 31 | BIOMOL2 | 1792 | F21 | 5mg/mL | EI270 | Rottlerin | inhibitor of p38 activated kinases | -1.777457 | 0 |
| 32 | BIOMOL2 | 1792 | M13 | 5mg/mL | G-236 | Manumycin A | ras farnesylation inhibitor | -1.755964 | 0 |
| 33 | BIOMOL2 | 1791 | N06 | 5mg/mL | GR301 | Camptothecin | topoisomerase 1 inhibitor, antineoplastic | -1.637238 | 0.151249 |
| 34 | BIOMOL2 | 1792 | L21 | 5mg/mL | EI-215 | Tyrphostin 9 | PDGF-R tyrosine kinase inhibitor | -1.622908 | 0 |
| 35 | BIOMOL2 | 1792 | E09 | 5mg/mL | GR307 | Etoposide | topoisomerase II inhibitor, antineoplastic | -1.568362 | 0 |
| 36 | NINDS | 1920 | M13 | 10 mM | 6004-24-6, 123-03-5 [anhydrous] | Cetylpyridinium chloride | antiinfective (topical) | -1.560866 | 0 |
| 37 | BIOMOL2 | 1791 | B10 | 5mg/mL | T-113 (Biomol) | Parthenolide | IkappaB kinase inhibitor | -1.552586 | 0 |
| 38 | BIOMOL2 | 1792 | I03 | 5mg/mL | GR-303 | Hoechst 33342 | DNA minor groove binder | -1.534591 | 0 |
| 39 | NINDS | 1922 | P16 | 10 mM | 35069-70-6 | 2,6-dimethoxyquinone | antibacterial, induces dermatitis, mutagen | -1.507747 | 0 |
| 40 | Prestwick | 1569 | B22 | 2 mg/ml | 316-42-7 | Emetine dihydrochloride | inhibits RNA, DNA and protein synthesis | -1.468808 | 0 |
| 41 | Prestwick | 1570 | M22 | 2 mg/ml | 66-81-9 | Cycloheximide | antibiotic. Protein synthesis inhibitor | -1.445816 | 0 |
| 42 | Prestwick | 1569 | E18 | 2 mg/ml | 6487-30-5 | Cephaeline dihydrochloride heptahydrate | nauseant alkaloid | -1.410059 | 0 |
| 43 | BIOMOL2 | 1791 | N11 | 5mg/mL | PI-110 | 3,4-dichloroisocoumarin | granzyme B inhibitor | -1.372644 | 0 |
| 44 | NINDS | 1923 | A13 | 10 mM | 34157-83-0 | Celastrol | antineoplastic, antiinflamatory, NO synthesis inhibitor, chaperone stimulant | -1.348443 | 0 |
| 45 | NINDS | 1920 | K12 | 10 mM | 316-42-7, 483-18-1 [emetine] | Emetine | inhibits RNA, DNA and protein synthesis | -1.288103 | 0 |
| 46 | Prestwick | 1568 | H21 | 2 mg/ml | 2112992 | **Camptothecin (S,+)** | topoisomerase 1 inhibitor, antineoplastic | -1.22608 | 0.109087 |
| 47 | NINDS | 1920 | M16 | 10 mM | 58-54-8 | Ethacrynic acid | diuretic | -1.226055 | 0 |
| 48 | NINDS | 1923 | C05 | 10 mM | 518-75-2 | Citrinin | antibacterial | -1.218574 | 0 |
| 49 | BIOMOL2 | 1792 | L07 | 5mg/mL | AP-300 | TPEN | cell permable heavy metal chelator | -1.18808 | 0 |
| 50 | Prestwick | 1569 | M11 | 2 mg/ml | 70476-82-3 | Mitoxantrone dihydrochloride | topoisomerase II inhibitor, antineoplastic | -1.170389 | 0 |
| 51 | NINDS | 1921 | N08 | 10 mM | 2112992 | Camptothecin | topoisomerase I inhibitor, antineoplastic | -1.152003 | 0.06008 |
| 52 | NINDS | 1922 | O21 | 10 mM | 70476-82-3, 65271-80-9 [mitoxantrone] | Mitoxanthrone hydrochloride | antineoplastic | -1.141116 | 0 |
| 53 | NINDS | 1920 | J10 | 10 mM | 19237-84-4, 19216-56-9 [prazosin] | Prazosin hydrochloride | antihypertensive | -1.098414 | 0 |
| 54 | NINDS | 1922 | O20 | 10 mM | 508-75-8 | Convallatoxin | cardiotonic | -1.088732 | 0 |
| 55 | Prestwick | 1568 | D20 | 2 mg/ml | 58-54-8 | Ethacrynic acid | Glutathione S-transferase inhibitor | -1.088665 | 9/E-7 |
| 56 | BIOMOL2 | 1792 | D17 | 5mg/mL | AC-146 | Prazocin | adrenoreceptor agonist | -1.074835 | 0 |
| 57 | BIOMOL2 | 1792 | B13 | 5mg/mL | CM109 | Ouabain | Na+K+ATPase inhibitor | -1.050767 | 0.000246 |
| 58 | NINDS | 1922 | H22 | 10 mM | 119413-54-6 | Topotecan hydrochloride | topoisomerase I inhibitor, antineoplastic | -1.033386 | 0 |
| 59 | BIOMOL2 | 1792 | N07 | 5mg/mL | KC-140 | Valinomycin | K+ ionophore | -0.984651 | 0 |
| 60 | NINDS | 1921 | D17 | 10 mM | 58-58-2, 53-79-2 [puromycin] | Puromycin hydrochloride | antineoplastic, antiprotozoal | -0.889753 | 0 |
| 61 | Prestwick | 1569 | O13 | 2 mg/ml | 33419-42-0 | Etoposide | topoisomerase II inhibitor, antineoplastic | -0.881592 | 0.003217 |
| 62 | Prestwick | 1571 | E13 | 2 mg/ml | 466-06-8 | Proscillaridin A | Na+/K+-ATPase inhibitor. Cardiac glycoside. | -0.876806 | 5/E-6 |

* The compounds listed are those shown below the red line in Figure 2A. Compounds highlighted in bold correspond to circled values.
